# Renal Papillary Necrosis Appearing as Bladder Cancer on Imaging

**DOI:** 10.1089/cren.2016.0011

**Published:** 2016-02-01

**Authors:** Lawrence M. Dagrosa, Fady Ghali, Elizabeth Ann Gormley

**Affiliations:** ^1^ Section of Urology, Dartmouth Hitchcock Medical Center, Lebanon, New Hampshire.; ^2^ Geisel School of Medicine at Dartmouth, Hanover, New Hampshire.

## Abstract

A 79-year-old woman with a history of diabetes mellitus and recurrent urinary tract infections (UTIs) presented with acute onset left lower quadrant pain, left-sided back pain, vomiting, and dysuria. Abdominopelvic CT scan revealed left hydroureteronephrosis to the level of the left ureterovesical junction (UVJ) where a bladder mass appeared to be obstructing the left ureteral orifice. The obstruction was ultimately found to be the result of a sloughed renal papilla lodged in the distal ureter, which created an inflammatory mass at the UVJ. Her history of diabetes and frequent UTIs likely predisposed her to the development of renal papillary necrosis (RPN) that resulted in sloughing of a renal papilla, distal ureteral obstruction with subsequent bladder inflammation that mimicked a bladder mass on imaging. RPN is a condition associated with many etiologies and likely represents a common final pathway of several diseases. Although several hypotheses exist, it is primarily thought to be ischemic in nature and is related to the underlying physiology of the renal papillae. We present a case of hydroureteronephrosis and bladder mass secondary to a sloughed renal papilla from RPN.

## Presentation of Case

**FIG. 1.**
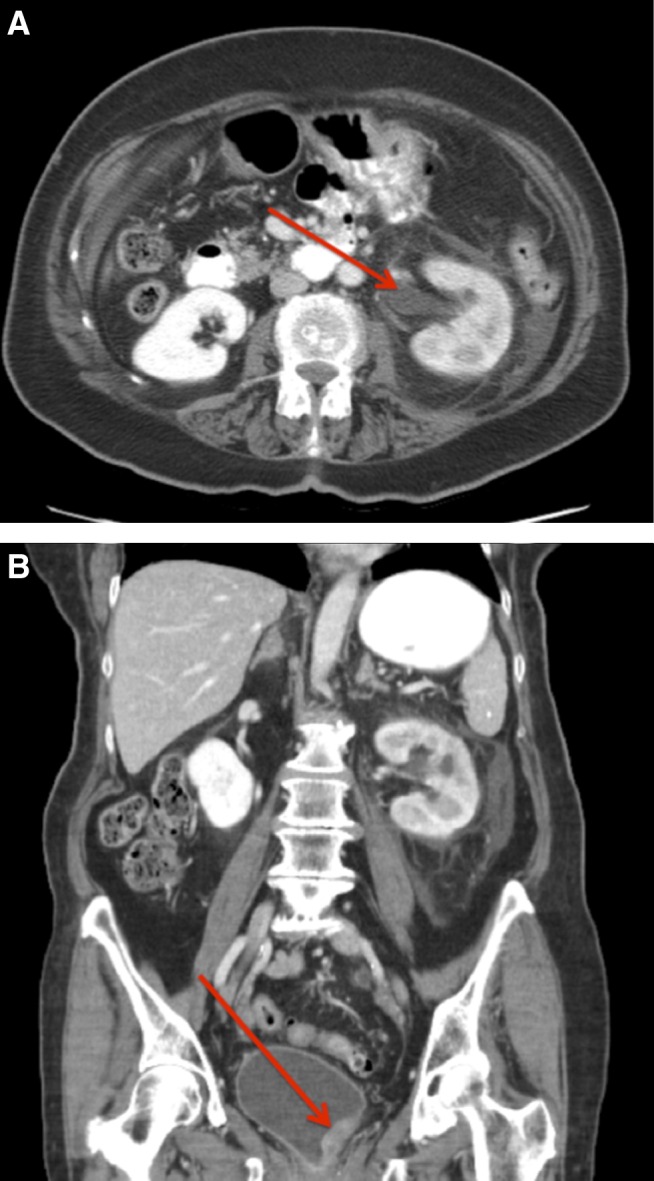
Abdominal and pelvic CT scan revealing left-sided hydroureteronephrosis and delayed nephrogram, suggesting obstruction **(A)** and bladder mass **(B)** appearing to obstruct the left ureteral orifice.

**FIG. 2.**
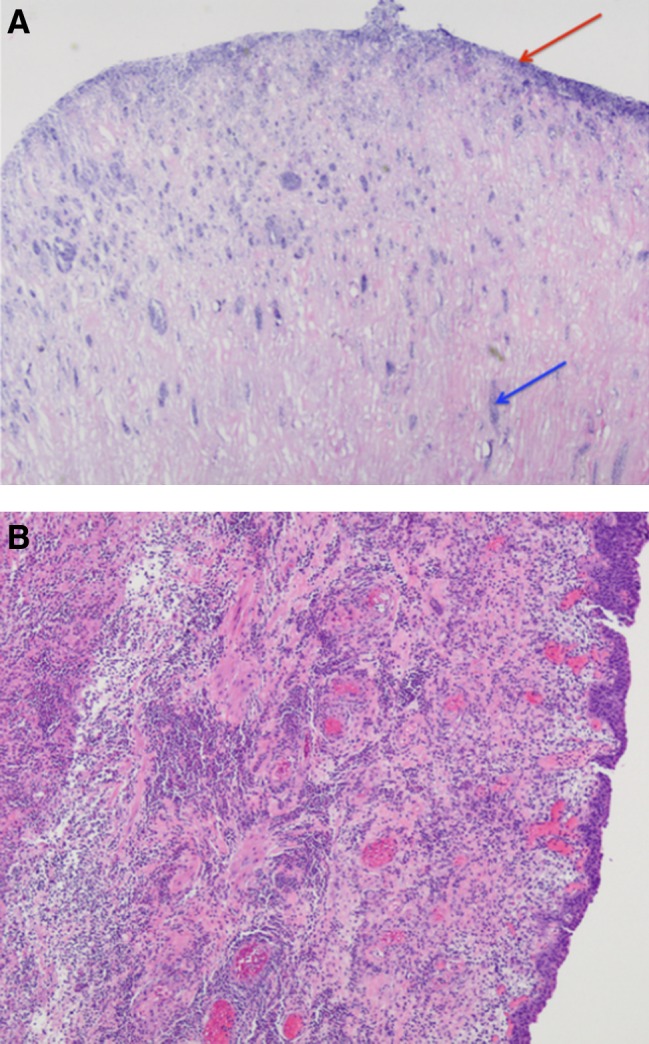
Final pathology analysis showing **(A)** duct-like structures filled with inflammatory and microbial cells (*blue arrow*) and microbial overgrowth (*red arrow*). **(B)** Sample of ureteral orifice showing diffuse inflammation with necrotic change and focal microabscesses.

A 79-year-old woman presented to the emergency department with 4 hours of acute onset, severe left lower quadrant pain associated with vomiting, dysuria, and chills. Her medical history was pertinent for type-2 diabetes mellitus and recurrent urinary tract infections (UTIs). She denied any smoking history. On evaluation, the patient was found to have left-sided costovertebral angle tenderness and left lower quadrant tenderness. She had a mild leukocytosis and pyuria, but a normal creatinine, estimated glomerular filtration rate, and was afebrile with normal heart rate and blood pressure. Imaging revealed left-sided hydroureteronephrosis (Fig. 1A) and a bladder mass (Fig. 1B). An invasive bladder cancer was suspected with resultant ureteral obstruction. The patient was brought to the operating room urgently to undergo transurethral resection of the mass and stenting of the left ureter. Intraoperatively the patient was found to have a mucous plug emanating from the mass. This was removed with a grasping forceps and further debris drained from the left ureteral orifice. The urothelium overlying a large bulge at the left ureteral orifice was grossly normal. Despite identifying the orifice and using a variety of wires, a guidewire or catheter could not be passed up the ureter. The left ureteral orifice and underlying tissue were resected and sent for pathology analysis, and a ureteral stent was placed. Pathology analysis of the mucous plug revealed necrotic linear duct-like structures with purulent inflammation and microbial overgrowth consistent with sloughed renal papilla (Fig. 2A). The tissue resected from the site of the ureteral orifice had diffuse inflammatory changes with focal necrotizing changes and a few microabscesses (Fig. 2B). Preoperative urine culture grew pan-sensitive *Escherichia coli* that was treated and the patient was discharged home the day after her surgery with stent removal on postoperative day 19. The patient's symptoms resolved after her procedure.

## Discussion and Literature Review

Renal papillary necrosis (RPN) is a condition that arises in the setting of various diseases and is characterized by diffuse ischemic necrosis of the renal medulla, particularly the papilla.^1,2^ Initially described in patients with long-standing diabetes, RPN was later found to have multiple etiologies.^2–4^ The mnemonic “POST CARDS” (Pyelonephritis, Obstruction, Sickle cell disease, Tuberculosis, Cirrhosis, Analgesic abuse, Renal vein thrombosis, Diabetes mellitus, Systemic vasculitis) is commonly used to illustrate the various causes of RPN.

The pathophysiology of RPN has not been fully elucidated and several competing hypotheses have been proposed. Ischemia has become the front-runner explanation of RPN despite early difficulties elucidating whether it was the cause or a result of this syndrome.^3^ Several animal studies, however, have demonstrated reduced vasa recta perfusion before the destruction of tubules,^5^ as well as platelet thrombi within the vasculature,^6^ helping establish ischemic events as a cause of RPN. The papillae are particularly susceptible to ischemic insults given the underlying renal vascular anatomy that supplies the renal papillae most distally through the tapering vasa recta. Indeed, even in the healthy individual, the papillae exist in a relative hypoxia because of the vascular arrangement and hypertonic environment of the renal medulla.^1^ Still, it is likely that several different pathways are at play depending on the underlying cause, and that RPN represents a final common pathway of multiple courses.^1,7–9^ Our patient had known diabetes that likely represented her biggest risk factor for RPN. Furthermore, her history of recurrent UTI raises the question of subclinical pyelonephritis preceding her presentation and further increasing her risk for papillary necrosis.

Early in its course, RPN often presents with nonspecific findings, including headaches and upper gastrointestinal symptoms. As it progresses, clinical signs may include irritative voiding symptoms, pyuria, hematuria, ureteral colic, lower back pain, and azotemia. Ultimately, these patients may develop hypertension, cardiac disease, and chronic renal failure.^3,7^ In this case, the patient presented with some of these symptoms: general malaise, gastrointestinal complaints, irritative voiding symptoms, and renal colic; however, her imaging was highly unusual as it appeared as an obstructing bladder mass most consistent with an invasive malignancy. As her RPN progressed, the sloughed debris obstructed her left ureter that resulted in renal colic. Furthermore, the accumulated debris caused a reactive inflammatory response in the underlying bladder tissue mimicking a mass within the bladder wall. To our knowledge, this is a unique presentation of RPN. Ultimately, removal of the obstructing sloughed papilla and surgical resection of the tissue from the ureterovesical junction with subsequent stenting and antibiotics resulted in relief of obstruction for our patient.

## Conclusion

Our case demonstrates that in patients at risk for RPN, sloughed debris can mimic urothelial tumors of the bladder on imaging.

## Abbreviations Used

CT: computerized tomography
RPN: renal papillary necrosis
UTI: urinary tract infection
UVJ: ureterovesical junction
